# International external quality assurance of *JAK2* V617F quantification

**DOI:** 10.1007/s00277-018-3570-8

**Published:** 2018-12-08

**Authors:** Julia Asp, Vibe Skov, Beatriz Bellosillo, Thomas Kristensen, Eric Lippert, Frank Dicker, Jiri Schwarz, Marzena Wojtaszewska, Lars Palmqvist, Susanna Akiki, Anni Aggerholm, Morten Tolstrup Andersen, François Girodon, Lasse Kjær, Elisabeth Oppliger Leibundgut, Alessandro Pancrazzi, Marta Vorland, Hajnalka Andrikovics, Robert Kralovics, Bruno Cassinat, Margarida Coucelo, Aleksandar Eftimov, Karl Haslam, Rajko Kusec, Dorota Link-Lenczowska, Laurence Lodé, Karolina Matiakowska, Dina Naguib, Filippo Navaglia, Guy Wayne Novotny, Melanie J Percy, Andrey Sudarikov, Sylvie Hermouet, Niels Pallisgaard

**Affiliations:** 1 Department of Clinical Chemistry and Transfusion Medicine, Institute of Biomedicine, the Sahlgrenska Academy, University of Gothenburg, Gothenburg, Sweden; 2 Department of Hematology, Zealand University Hospital, Roskilde, Denmark; 3 Department of Pathology, Hospital del Mar, Barcelona, Spain; 4 Department of Pathology, Odense University Hospital, Odense, Denmark; 5 CHU de Brest, Brest, France; 6 Munich Leukemia Laboratory, Munich, Germany; 7 Institute of Hematology and Blood Transfusion, Prague, Czech Republic; 8 Department of Hematology and Bone Marrow Transplantation, Poznan University of Medical Sciences, Poznan, Poland; 9 Department of Laboratory Medicine and Pathology, Qatar Rehabilitation Institute (QRI), Hamad Bin Khalifa Medical City (HBKM), Doha, Qatar; 10 Aarhus University Hospital, Aarhus, Denmark; 11 Rigshospitalet, Copenhagen, Denmark; 12 CHU Dijon/INSERM U866, Dijon, France; 13 University Hospital Bern and University of Bern, Bern, Switzerland; 14 Centro di Ricerca e Innovazione per le Malattie Mieloproliferative (CRIMM), Florence, Italy; 15 Haukeland University Hospital, Bergen, Norway; 16 Central Hospital of Southern Pest, Budapest, Hungary; 17 CeMM Research Center for Molecular Medicine of the Austrian Academy of Sciences, Vienna, Austria, and Department of Internal Medicine I, Medical University of Vienna, Vienna, Austria; 18 Service de Biologie Cellulaire, AP-HP, Hopital Saint-Louis, Paris, France; 19 Clinical Hematology Unit, Hospital Pediátrico, Centro Hospitalar e Universitário de Coimbra, Coimbra, Portugal; 20 Center for Biomolecular Pharmaceutical Analyses, Faculty of Pharmacy, UKiM, Skopje, Republic of Macedonia; 21 St James’s Hospital, Dublin, Ireland; 22 Dubrava University Hospital and Zagreb School of Medicine, University of Zagreb, Zagreb, Croatia; 23 Molecular Diagnostics Laboratory, Hematology Diagnostics Department, Jagiellonian University Hospital, Krakow, Poland; 24 Hématologie Biologique, CHRU de Montpellier, Montpellier, France; 25 Faculty of Medicine, Nicolaus Copernicus University in Torun, Bydgoszcz, Poland; 26 CHU Côte de Nacre, Caen, France; 27 Department of Laboratory Medicine, University - Hospital of Padova, Padova, Italy; 28 Department of Hematology and Department of Pathology, Molecular Unit, Herlev Hospital, University of Copenhagen, Herlev Ringvej 75, DK-2730 Herlev, Denmark; 29 Belfast City Hospital, Belfast, UK; 30 National Research Center for Hematology, Moscow, Russia; 31 Laboratory of Hematology, University Hospital (CHU) Nantes, Nantes, France; 32 CRCINA, Inserm UMR892 / CNRS UMR6299, Centre de Recherche en Cancérologie et Immunologie Nantes-Angers, Université de Nantes, Nantes, France

**Keywords:** *JAK2* V617F, External quality assurance, Myeloproliferative neoplasms, Quantitative PCR

## Abstract

External quality assurance (EQA) programs are vital to ensure high quality and standardized results in molecular diagnostics. It is important that EQA for quantitative analysis takes into account the variation in methodology. Results cannot be expected to be more accurate than limits of the technology used, and it is essential to recognize factors causing substantial outlier results. The present study aimed to identify parameters of specific importance for *JAK2* V617F quantification by quantitative PCR, using different starting materials, assays, and technical platforms. Sixteen samples were issued to participating laboratories in two EQA rounds. In the first round, 19 laboratories from 11 European countries analyzing *JAK2* V617F as part of their routine diagnostics returned results from in-house assays. In the second round, 25 laboratories from 17 countries participated. Despite variations in starting material, assay set-up and instrumentation the laboratories were generally well aligned in the EQA program. However, EQA based on a single technology appears to be a valuable tool to achieve standardization of the quantification of *JAK2* V617F allelic burden.

## Introduction

The discovery of the c.1849G>T mutation leading to the p.Val617Phe (V617F) substitution in *JAK2* [1–4] has been a landmark in molecular diagnosis of the myeloproliferative neoplasms (MPN) polycythemia vera (PV), essential thrombocythemia (ET), and primary myelofibrosis (PMF). Quantification of the mutation has shown that mutation burden also could reflect different subtypes of MPN. The majority of patients with PV or fibrotic PMF have been reported to have more than 50% *JAK2* V617F while the opposite has been seen in ET patients [5, 6]. In addition, quantification of the allelic burden in *JAK2* V617F-positive patients is increasingly used to monitor treatment response of new targeted therapies as well as in transplanted patients [7–9].

For molecular diagnosis, it has been recommended that the assay should be sensitive enough to detect a mutant burden around 1% [10]. The combination of a sensitive detection and reproducible quantification of *JAK2* V617F challenges the methodology used in a routine setting. Conventional Sanger sequencing does not show the required sensitivity in cases with low mutation burden, and methodologies involving next generation sequencing are unnecessarily labor intensive and expensive for mutation detection of a single nucleotide substitution. Instead, the use of quantitative polymerase chain reaction (qPCR) has been shown to be a both sensitive and cost-effective method [11] and superior in sensitivity compared to qualitative methods [12]. As a step towards standardization of reliable molecular diagnostics, the European Leukemia Net (ELN) and MPN&MPNr-EuroNet have evaluated performance of different allele-specific (AS)-qPCR assays [8]. This work, involving 12 laboratories from seven countries recommended a *JAK2* V617F qPCR assay which showed consistent performance across different qPCR platforms [13]. Even so, variation between laboratories and different instrumental setups can be substantial despite the use of the same experimental protocol. To ensure high quality and standardized quantitative results, external quality assurance (EQA) programs are vital. A program dedicated to *JAK2* V617F detection by qPCR is advantageous since no additional bias on quantification would be introduced by comparison to a different methodology. MPN&MPNr-EuroNet has performed two rounds of EQA based on qPCR assays. In addition to providing an EQA to participating laboratories in the network, the aim was to identify parameters critical for the quantification of *JAK2* V617F. Such factors would have a substantial impact also on an EQA result, and thus need to be identified in order to design a beneficial EQA program which would be useful in clinical routine.

## Materials and methods

### Participants

For the first quality assurance round (QA1), 19 laboratories from 11 countries across Europe analyzing *JAK2* V617F by qPCR as part of their routine diagnostics returned results obtained with in-house assays. In the second QA (QA2), 25 laboratories from 17 countries participated.

### Samples and references

Blood samples from *JAK2* V617F-positive patients were collected after informed consent according to the guidelines of the Danish Regional Science Ethics Committee. In QA1, ten blood samples were collected, aliquoted, and distributed to participating laboratories by an overnight courier. DNA was extracted locally from whole blood according to each participant’s standard procedure. Six participants received extra blood and extracted DNA also from hemolyzed blood (*n* = 3) or granulocytes (*n* = 3) in addition to whole blood. In QA2, six unknown samples prepared by spiking *JAK2* V617F-positive HEL cell line DNA into normal wild-type donor DNA was sent out. In both QA1 and QA2, a common reference for calibration corresponding to 75%, 23%, 3%, and 0.3% *JAK2* V617F was created by spiking a 648 bp PCR fragment containing the c.1849G>T mutation into normal wild-type donor DNA and distributed with the samples. Droplet digital PCR (ddPCR, Bio-Rad, Hercules, CA, USA) was used to obtain a reference value for each sample in the trials by taking the mean of four replicates repeated three times. In QA2, values obtained by ddPCR in a separate laboratory were added to the mean as well.

### Quantification of JAK2 V617F by qPCR

Copy numbers for *JAK2* V617F and *JAK2* WT and the allelic ratios of *JAK2* V617F expressed as % [*JAK2* V617F copy number/(*JAK2* WT copy number + *JAK2* V617F copy number)] were determined by the participating laboratories according to the assay used in the clinical routine. All results were sent to one laboratory for further analysis. To determine general variation of qPCR within an assay, data was collected from control samples and repeatedly analyzed according to the Larsen protocol [13] during 12 months in one laboratory. The analysis was performed by different persons on two PCR instruments, and batches for reagents were changed during the 12-month period. Percentage *JAK2* V617F was calculated for each sample and the coefficient of variation (CV) for the assay was determined.

## Results

### Similar EQA results with different starting materials, qPCR assays, and qPCR instruments

To identify the parameters of specific importance for causing outliers in a *JAK2* V617F EQA where a quantitative value of mutation burden is determined by qPCR, different starting materials, different qPCR assays, and different technical platforms were included. In total, 16 samples with unknown mutation burden were issued to participating laboratories. In QA1, samples were divided into four groups based on the reference levels of *JAK2* V617F as determined by ddPCR: < 2% (*n* = 4), 2–10% (*n* = 3), 10–20% (*n* = 2), and > 30% (*n* = 2). Results were analyzed in detail for one sample in each group.

**Fig. 1:**
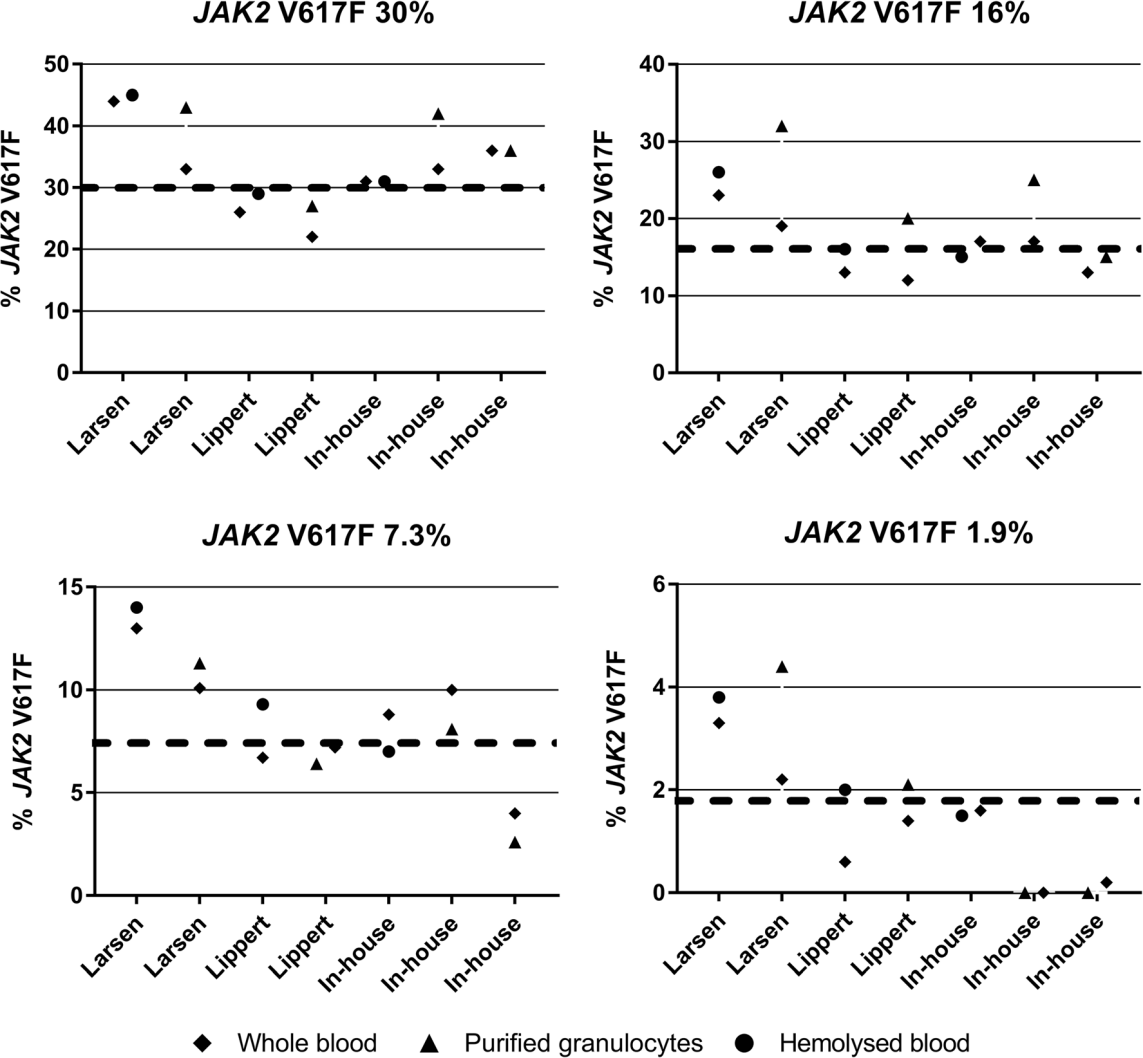
Comparison of different starting materials for quantitative analysis of *JAK2* V617F on selected samples. *JAK2* V617F detection was performed in parallel using different starting materials in samples with four different levels of *JAK2* V617F mutation. One of the six participating laboratories analyzed the samples with two different assays yielding a total of seven sets of data. Assigned values of *JAK2* V617F are reference values as determined by ddPCR. These are indicated in the graphs by headings and dotted lines

To test starting material for the analysis, six different laboratories extracted DNA from purified granulocytes or hemolyzed blood in addition to whole blood. *JAK2* V617F was analyzed from both types of starting materials in parallel using routine protocol(s). Although differences could be noted between starting materials when comparisons were made within the same laboratory, the difference was in the same range as between the laboratories and different assays (Fig. 1).

To study the influence of assay protocols on EQA results, 19 laboratories from 11 countries analyzing *JAK2* V617F by qPCR as part of their routine diagnostics returned results from their assay protocol used in clinical diagnostics in QA1. One of the laboratories returned results from two different assays yielding 20 sets of data in total. Various qPCR assay protocols were used: Larsen [13], *n* = 6; Lippert [5], *n* = 5; Ipsogen Mutaquant kit (Qiagen, Marseille, France), *n* = 4; and other protocols (in-house assays), *n* = 5. Although reported copy numbers in samples varied between laboratories (data not shown), the % *JAK2* V617F was rather consistent across different assays (Table 1). In QA2, 25 laboratories from 17 countries returned results. Two of the laboratories returned results from two different assays yielding 27 sets of data. In QA2, the majority of participating laboratories used the Larsen assay (*n* = 18) or a modification of this assay (*n* = 4). Five laboratories reported results obtained by another assay. The six samples issued in QA2 were divided into the same groups as for QA1 (< 2% (*n* = 2), 2–10% (*n* = 1), 10–20% (*n* = 2), and > 30% (*n* = 1)) and one sample from each group was analyzed in detail. Overall, variations were similar in QA1 and in QA2 (Table 1). Although there was a relative consistency in quantification of *JAK2* V617F allelic burdens above 2%, a higher variation was noted in samples with low mutation burden (< 2%).

**Table 1:** % *JAK2* V617F obtained using different qPCR assays

EQA1	Larsen (*n* = 6)		Lippert (*n* = 5)		Ipsogen (*n* = 4)		Other* (*n* = 5)	
ddPCR %	Mean %	CV%	Mean %	CV%	Mean %	CV%	Mean %	CV%
30	31	24	31	25	34	10	29	24
16	18	24	17	31	22	19	15	28
7.3	8.6	31	9.9	36	10	21	7.1	41
1.9	2.1	34	1.7	44	2.5	39	0.8	89
EQA2	Larsen incl. modified (*n* = 22)						Other* (*n* = 5)	
ddPCR %	Mean %	CV%					Mean %	CV%
66	61	14					65	17
22	19	32					22	46
4.6	4.0	44					4.5	51
1.0	0.7	34					0.8	37

*The “other” group does not include the same laboratories and protocols in EQA1 and EQA2

**Fig. 2:**
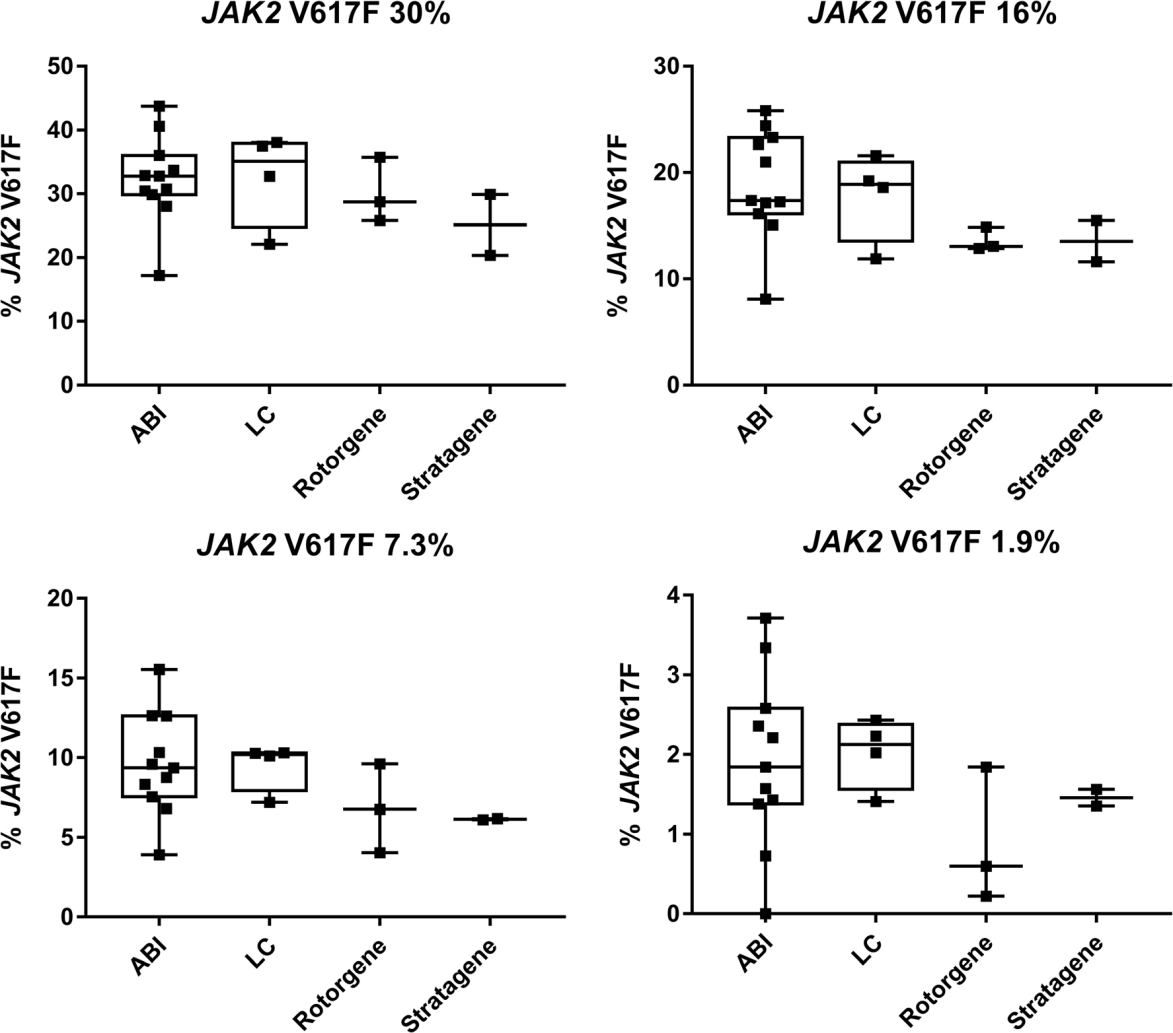
*JAK2* V617F results obtained with different qPCR instruments on selected samples. Different versions of instruments were included in each group according to text. ABI *n* = 11, LC *n* = 4, Rotorgene *n* = 3, and Stratagene *n* = 2. Median values in each group are indicated by a black line in boxes. Assigned values of *JAK2* V617F are reference values as determined by ddPCR. These are indicated in the graphs by headings

Next, we studied whether different qPCR platforms could introduce substantial variation. The majority of QA1 participants used instruments from Applied Biosystems (Foster City, CA, USA). Eleven sets of data were analyzed on these instruments (ABI7300/7500/7500FAST/7900HT). The remaining laboratories used Lightcycler LC480 (Roche Applied Science, Penzberg, Germany, *n* = 4), Rotorgene (3000A/Q; Corbett Life Science, Sydney, Australia; Qiagen, *n* = 3), or Stratagene (MX3000/MX3500; Agilent Technologies, Santa Clara, CA, USA, *n* = 2) for analysis. For all but Applied Biosystems instruments, groups were very small, which resulted in single outliers having a substantial impact on the results. In addition, different versions of instruments from the same manufacturer were used in all groups. Nonetheless, no major difference depending on qPCR instrument could be seen (Fig. 2).

**Fig. 3:**
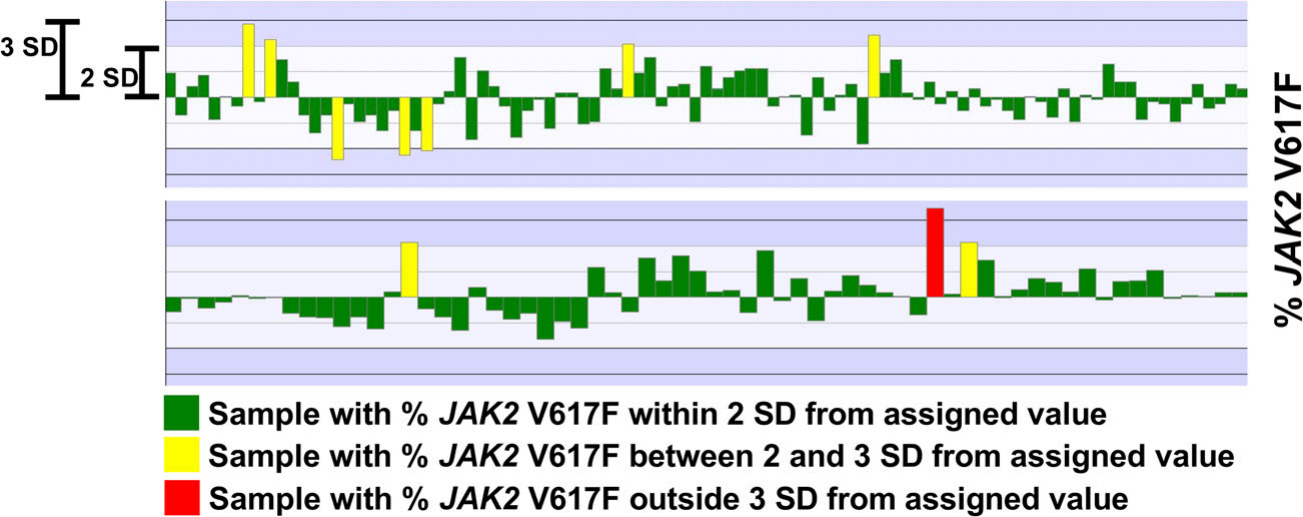
Variation in control samples. Repeated analysis of control samples were performed during a 1-year period. The upper panel shows results from a sample with 4.5% *JAK2* V617F; the lower panel shows results from a sample with 13% *JAK2* V617F. SD was 1.2 and 3.5 respectively

For comparison, CV for the Larsen assay over a stretch of one year was determined in one participating laboratory. During that period of time, a control sample of 4.5% *JAK2* V617F was analyzed 97 times and a sample of 13% was analyzed 64 times on two instruments (Rotorgene Q, Fig. 3). CV for calculated % *JAK2* V617F was 26% in both cases.

To evaluate whether the differences between assays and qPCR instruments were substantial enough to affect the result of an EQA, z-scores were determined for selected samples in QA1. A z-score between 2 and − 2 was considered as satisfactory performance, a z-score between 2 and 3 or between − 2 and − 3 was considered as a warning, and a z-score above 3 or below − 3 was considered as critical. Results showed that three participants obtained a warning, while the remaining participants got a satisfactory performance. None was scored as “critical” (Table 2).

**Table 2:** Z-scores for selected *JAK2* V617F samples

	z-score*							
ddPCR % *JAK2* V617F	< − 3.0	− 3.0–− 2.0	− 2.0–− 1.0	− 1.0–0	0–1.0	1.0–2.0	2.0–3.0	> 3.0
30		1	2	7	7	3		
16			3	8	5	4		
7.3			2	8	7	2	1	
1.9			4	6	8	1	1	

*Frequency of participants with each z-score indicated in table

## Discussion

Bias altering qPCR results may occur at several steps of *JAK2* V617F assays, even when laboratories use the same methodology. Starting material for the analysis as well as technical platform, assay design and batch variations can influence the results. Even among laboratories using the same qPCR protocol for quantitative assays, considerable variation has been reported [14]. Standardized results are vital not only to aid in diagnosis of patients but also in clinical, multicenter studies. One way to test how well individual laboratories align to predicted results is through participating in EQA. Moreover, EQA are central tools for the accreditation and assessment of laboratory performance. To design a useful EQA for quantitative analysis, it is important to take into account the variation of the methodology in focus. If expectations of consistency in results are set too high, beyond the limits of the technology used, there is a risk that a well-performing laboratory will get poor or inadequate results just because of natural variation in the method, or because of the influence of a particular parameter which has not been identified as important for outcome. Therefore, it is essential to recognize factors which would cause substantial outliers in the tests, as well as which variation could be expected from different qPCR technical platforms.

A previous study has shown that the results obtained for the detection of the *JAK2* mutation were comparable in whole blood and in purified granulocytes, and that no false negative was reported in whole blood if the qPCR assay used was able to detect < 1% *JAK2* V617F [15]. However, in this study, the allelic ratio was reported to be on average 15% lower in whole blood than in purified granulocytes; the low-average *JAK2* V617F values was due to a minority of the whole blood samples. The choice of the starting material could thus be of importance in individual cases depending on the question asked. In the present study, the starting material used for the analysis did not affect the performance in EQA for the majority of laboratories.

In both QA1 and QA2, samples with low mutation burden (< 2% *JAK2* V617F) were included, and a greater variation was seen for these samples. This reflects the sensitivity of the assay and the qPCR setup in each laboratory. In addition, when dealing with low *JAK2* V617F copy numbers stochastic variation will add to the overall variation. However, for low mutation burden, specificity of the assay is an equally important issue. The background level where cross-reaction with the wild-type allele could occur must be clearly defined by each laboratory to avoid false positive results [10].

To be able to compare results, over time as well as between laboratories, there is a need to standardize the results with respect to the quantitative level of mutation burden. In chronic myeloid leukemia, where the level of expression of the fusion gene *BCR-ABL1* is correlated to prognosis, a conversion factor has been established to correct for differences across laboratories. This factor is used to align results to an international scale which is anchored to clinical results [16, 17]. However, the original conversion factor was based on the sample exchange with a reference laboratory and this procedure is both time-consuming and expensive and a risk for inborn errors due to bias cannot be ruled out. To overcome this, primary references intended for the calibration of a secondary reference material have been established [18]. In addition, a certified reference plasmid for the calibration of *BCR-ABL1* quantification has been manufactured [19]. As reported in a previous international study [11], a common reference material remains a useful tool for laboratories also for *JAK2* V617F, as it allows decreasing or suppressing differences in copy numbers in certain laboratories. In addition, it also allows adjustment for batch variations, e.g., due to differences in quality of primer oligonucleotides. A first WHO reference panel for *JAK2* V617F has recently been established and is now available [20]. This holds promise to further improve assay standardization. With increasing clinical demands for molecular monitoring, both EQA programs and standardized *JAK2* V617F reference material are needed to identify and maintain validated laboratories.

In conclusion, variation in method due to the starting material, assay set-up, or qPCR equipment did not result in significant outliers in the EQA programs included in this study. However, EQA based on a single technology remains a valuable tool to achieve standardization of *JAK2* V617F quantification.
